# Sequenced psychotherapy improves evidence-based trauma-focused psychotherapy initiation and retention in a national sample of veterans

**DOI:** 10.1016/j.psychres.2025.116612

**Published:** 2025-06-24

**Authors:** William R. Wolfe, Anna Staudenmeyer, Marylene Cloitre, Asale Hubbard, Martha Schmitz, Brian Mohlenhoff, Shira Maguen, Thomas C. Neylan

**Affiliations:** a Department of Psychiatry and Behavioral Sciences, University of California, San Francisco, 675 18th St., San Francisco, CA 94107, USA; b San Francisco Veterans Affairs Health Care System, 4150 Clement St (116P), San Francisco, CA 94121, USA; c New York University Silver School of Social Work, One Washington Square North, New York, NY 10003, USA

**Keywords:** Posttraumatic Stress Disorder, Cognitive Processing Therapy, Prolonged Exposure Therapy, Veterans Affairs, Psychotherapy, Phase-Based Psychotherapy, Sequenced Psychotherapy

## Abstract

**Background:**

Posttraumatic stress disorder (PTSD) is a disabling condition costing the Veterans Administration (VA) $5 billion annually in disability compensation. Despite system-wide dissemination of Cognitive Processing Therapy (CPT) and Prolonged Exposure (PE) since 2007, only a small minority of veterans complete these treatments. We investigated the impact of sequenced treatment on initiation of and retention in CPT and PE across self-reported race and ethnicity, as well as recent heavy drinking and suicidal ideation (SI).

**Methods:**

VA administrative data were used to assess the impact of sequenced psychotherapy (SP), in which ≥ 8 sessions of non-trauma-focused individual (SPI) or group (SPG) psychotherapy was delivered before trauma-focused care, on initiation and retention in CPT and PE over two years from PTSD treatment initiation. Results were analyzed by self-reported race and ethnicity, heavy drinking (AUDIT-C ≥6), and SI (PHQ9 Q9>1).

**Results:**

Nationwide, 12.9 % of veterans who entered care for PTSD between 10/1/2014 and 11/30/2020 (n = 490,097) initiated VA-disseminated evidence-based treatment within 21 months (9.5 % CPT, 3.4 % PE). Among those, treatment retention (≥ 8 sessions) was 46.4 % and 42.3 %, respectively. SPI and SPG were associated with 0.4-6.8 % increases in CPT and PE initiation across racial/ethnic and risk groups. SPI was associated with increased CPT and PE retention of 8.0 % and 8.2 %; for SPG, retention increases were 3.4 % and 8.7 %. Strikingly, Hispanic White veterans with heavy drinking had 21.7 % increased CPT retention following SPG.

**Conclusions:**

Sequential individual and group psychotherapy may improve initiation and completion of CPT and PE, particularly for certain minoritized and high-risk groups.

## 1. Introduction

Posttraumatic stress disorder (PTSD) is a costly and disabling chronic mental health condition which has a profoundly negative impact on quality of life and overall functioning throughout the lifespan. In 2024, the U.S. Department of Veterans Affairs (VA) paid an estimated $5.4 billion in PTSD disability compensation (U.S. Department of Veterans Affairs, 2023a, 2023b, 2024). The VA has invested heavily in system-wide efforts since 2007 to disseminate Cognitive Processing Therapy (CPT) and Prolonged Exposure (PE) for the treatment of PTSD. Despite the strong clinical trial evidence supporting these Trauma-Focused Evidence-Based Psychotherapies (TF-EBPs), treatment initiation and retention have fallen far short of expectations. A recent study found that of veterans receiving a diagnosis of PTSD between 2017 and 2019, only 11.6 % initiated a TF-EBP in their first year of treatment, and of those, 67 % dropped out (Cameron et al., 2023). Consequently, the burden of inadequately treated PTSD among veterans remains unacceptably high.

The challenges of addressing this treatment burden are compounded by the complexity of the veteran clinical population, which includes risk factors such as heavy drinking and suicidal ideation and demonstrates racial and ethnic heterogeneity. We are aware of only one study comparing TF-EBP retention rates among veterans with and without alcohol use disorder (Kaysen et al., 2014), which found no difference in number of CPT sessions completed among 536 veterans with PTSD. Nevertheless, concern about treatment dropout in this population has been high, with specialized treatments developed for use prior to trauma-focused care (Najavits et al., 1998; Norman et al., 2010) or integrated with it (Mills et al., 2012). Similarly, suicidal ideation (SI) is highly prevalent and poses significant risk to PTSD treatment populations (Akbar et al., 2023; Arenson et al., 2018; Sala-Hamrick et al., 2023). Although we are aware of no studies comparing retention in TF-EBP between groups with and without SI, there is evidence that TF-EBPs are effective in reducing SI among individuals with PTSD, and that improvements in PTSD symptoms are associated with decreases in SI (Brown et al., 2019; Johnson et al., 2021). Thus, TF-EBP initiation and retention should be a high priority for this group. Finally, there is evidence that barriers to treatment initiation and retention are unevenly distributed among different racial and ethnic groups. While two studies of civilian PTSD samples found that Black women were less likely to initiate CPT then White women (Iverson et al., 2011; Lester et al., 2010), in a study of post-9/11 veterans with PTSD, Black and Hispanic female veterans were significantly less likely to receive minimally adequate care (defined as 9 or more psychotherapy visits in a 15-week period or at least 12 consecutive weeks with a first-line medication) compared to their White female counterparts within a year of initiating treatment (Hebenstreit et al., 2015). Another large cohort of returning veterans from post-9/11 deployment found that Hispanic veterans were less likely to complete CPT than non-Hispanic veterans (Maguen et al., 2019).

Strategies for improving treatment retention across demographic and risk populations have included transitioning to a higher level of care (residential treatment) or increasing visit frequency (“massed treatment”). A recent meta-analysis found that individuals were more likely to complete outpatient trauma-focused treatment when it was delivered at a higher frequency (Hoppen et al., 2023). Although promising, these strategies can present barriers to broad dissemination based on cost, provider scheduling constraints, and increased scheduling burden for veterans, limiting access for those with academic, caregiving, or work-related commitments. Another strategy for improving treatment retention is to provide sequenced psychotherapy (SP), in which non-trauma-focused individual or group psychotherapy is delivered *before* initiating a TF-EBP. Although research suggests that VA providers often consider veteran readiness when offering TF-EBPs (Cook et al., 2017; Finley et al., 2020; Hamblen et al., 2015; Rosen et al., 2016), sequenced psychotherapy remains controversial, with some arguing that this strategy delays access to effective treatments and diverts veterans from initiating TF-EBPs without offering any benefit for PTSD care. Attempts to resolve this question using data from real-world VA PTSD clinics have been limited. Two studies refute the notion that group SP builds readiness for TF-EBP (Dedert et al., 2021; Wiedeman et al., 2020), while findings from our PTSD clinic, which offers individual and group sequenced treatment, showed that individual SP increased TF-EBP completion rates from 50 % to 93 % (Staudenmeyer et al., 2022).

Naturalistic clinic-based studies are constrained by limitations in scope and sample size. Conducted in PTSD specialty settings, these studies do not account for psychotherapy provided prior to referral, rendering those treatments invisible to the analysis. Also overlooked in these studies are veterans not referred for PTSD specialty care, whether because of concern about lack of readiness for TF-EBP or based on specific referral criteria of the target clinics. Finally, these studies lack sufficient sample size to assess key variables related to sequenced treatment, such as differential effects among demographic groups or high-risk subpopulations.

Maguen et al. (2019) used a national dataset of returning combat veterans from Iraq and Afghanistan for a cross-clinic analysis, finding that prior psychotherapy was a significant predictor of CPT and PE completion (Maguen et al., 2019). The current study builds on that approach by broadening to veterans of all eras. We utilized administrative data from 130 Veterans Affairs healthcare systems to include all veterans entering VA mental health treatment for PTSD between October 2014 and November 2020. We sought to investigate how an initial course of individual and group psychotherapy impacted the likelihood of subsequently initiating and completing CPT and PE, and whether results differed across self-reported racial and ethnic groups. We identified veterans with heavy drinking and frequent suicidal ideation (SI), factors often considered when evaluating readiness for TF-EBPs (Back et al., 2009; Cook et al., 2017; Minnen et al., 2002; Tarrier et al., 2000), to assess differences in TF-EBP initiation and retention and to understand the role that sequenced treatment may play in TF-EBP retention among these higher acuity groups. We hypothesized that SP would improve CPT and PE retention without reducing initiation of these evidence-based treatments, and that these effects might differ among race/ethnicity and risk groups.

## 2. Methods

### 2.1. Sample

The national sample of 490,097 veterans was drawn from 130 Department of Veterans Affairs healthcare systems providing outpatient mental health services.

### 2.2. Procedure

Using the VA Corporate Data Warehouse (CDW), a national repository of VA electronic medical records, we included all veterans who entered treatment for PTSD (defined as having ≥ 3 mental health visits coded with a primary diagnosis of PTSD following ≥ 18 months without mental health visits) between 10/1/2014 and 11/30/2020 (n = 490,097). We used a 2-year follow-up from treatment entry to identify sequenced psychotherapy (SP) visits and CPT/PE visits. These visit types were identified using Current Procedural Terminology codes (CPT codes) and VA national mental health Evidence-Based Psychotherapy (EBP) templates; primary diagnosis was identified using International Classification of Diseases (ICD)-9 and ICD-10 codes for PTSD.

VA categories of self-reported race and ethnicity were utilized, combining six racial categories (Non-Hispanic White [NHW], Black, Asian, American Indian/Alaska Native, Native Hawaiian/Other Pacific Islander, and Multiracial), with a separate ethnicity category (Hispanic vs. non-Hispanic). Veterans who identified their ethnicity as Hispanic and their race as White were grouped as “Hispanic White” (HW); because of sample size constraints, other Hispanic veterans were grouped according to race only. HW veterans, together with veterans who self-identified as belonging to races other than White were grouped as Black, Indigenous, People of Color (BIPOC) for comparison to the NHW group.

Alcohol use was measured with the Alcohol Use Disorders Identification Test-Consumption (AUDIT-C), a three-item screening tool for risky alcohol consumption (Conigrave et al., 1995). Scores range from 0 to 12, with scores of 4 or more in men (3 or more in women) typically indicating risky drinking behavior. Heavy Drinking was defined as any score of ≥ 6 in the prior 36 months; it was compared with Light Drinking (maximum score ≤ 3; range 0-12). AUDIT-C scores for veterans identified as female were increased by one point in our analysis to adjust for the gender-based difference in the threshold for risky drinking.

SI was identified using Item 9 on the Patient Health Questionnaire (PHQ-9) (Kroenke et al., 2001): “thoughts you would be better off dead, or of hurting yourself.” We included veterans who, at any time from 12 months before to 1 month after entering treatment, endorsed frequent SI: having these thoughts *more than half the days* or *nearly every day* over the past two weeks, scored as a 2 or 3 on item 9.

### 2.3. Variables

#### 2.3.1. Sequenced psychotherapy

Sequenced psychotherapy (SP) was coded according to the format (individual vs. group) of each session, namely, whether the psychotherapy preceding CPT or PE was individual (SPI) or group (SPG). The dose for the initial therapy was defined as the maximum number of outpatient mental health encounters in any 12-week period that used psychotherapy CPT codes and occurred before initiation of CPT or PE; if neither of these treatments was initiated, all qualifying visits that occurred during the follow-up period were included. An episode of the initiating psychotherapy in SP was defined as ≥ 8 sessions in 12 weeks, based on previous research establishing similar cutoffs for minimally adequate mental health treatment (Maguen et al., 2012; Seal et al., 2010; Sripada et al., 2016; P. S. Wang et al., 2000).

#### 2.3.2. CPT and PE visits

CPT and PE sessions were identified using health factors associated with the mandatory EBP templates (“MH CPT THERAPY” for individual CPT and “MH PEI THERAPY” for PE sessions). CPT sessions conducted in group format were not associated with mandatory templates and were therefore excluded; references to CPT sessions throughout this text denote individual CPT, in which the CPT template was used for a session coded as individual psychotherapy. Using this method, CPT and PE sessions were counted for the interval ending two years after treatment entry. Initiation was defined as attending ≥ 1 session of CPT or PE. Retention was defined as attending ≥ 8 sessions of CPT or PE within any 24-week period, a conservative cutoff established in previous research (Maguen et al., 2019). If a veteran engaged in both CPT and PE during the study period, only one of these treatments was included in the analysis, with preference given to treatments in which the retention criterion was met (**≥** 8 sessions) or those with the most sessions. If a veteran had ≥ 8 sessions of both CPT and PE, the first completed treatment was used in the analysis. Dropout was defined as attending ≤ 7 sessions of CPT or PE. In order to reduce “false dropouts” for treatments initiated near the end of the 24-month follow-up period, CPT or PE sessions which began 21-24 months after treatment entry were excluded from the analysis.

### 2.4. Data Analysis

Chi-square analyses compared the likelihood of CPT or PE retention among veterans with and without SP, and p < .001 was considered statistically significant. Comparisons were between: a) veterans who completed ≤ 1 SPI visit and those who completed ≥ 8 SPI visits in 12 weeks; b) veterans who completed ≤ 1 SPG visit and those who completed ≥ 8 SPG visits in 12 weeks. Changes in percent retention is reported between comparison groups.

## 3. Results

### 3.1. CPT / PE initiation and retention rates

Table 1 lists CPT / PE initiation and retention rates for the study sample. Of the 490,097 veterans who entered mental health treatment for PTSD during the 74-month study period, only 46,411 (9.5 %) initiated CPT and 16,556 (3.4 %) initiated PE within 21 months of entering mental health treatment. CPT and PE initiation rates for BIPOC veterans did not appear to be meaningfully different from those of NHW veterans. Among those veterans in our sample who initiated CPT or PE, overall retention rates were 46.4 % and 42.3 %, respectively. While there were not large differences between racial and ethnic groups in initiation rates, NHW veterans were 4.1 % more likely to be retained in CPT (*p <* .001) and 5.1 % more likely to be retained in PE (*p <* .001) compared to HW veterans, and 10.6 % more likely to be retained in CPT than Pacific Islander veterans (*p <* .001). AUDIT-C data from within the previous 18 months, used to identify heavy vs. light drinking, were available for 85 % of veterans in the sample. Although there was little difference in CPT and PE initiation between heavy drinkers and light drinkers, heavy drinking appeared to increase dropout from CPT (3.1 %, p < .001) and PE (4.0 %, p < .001). PHQ9 data from within the prior 12 months, used to identify veterans vs. without SI, were available only for 23 % of veterans in the sample. There was little difference in CPT and PE initiation and no difference in CPT and PE retention for veterans with SI compared to those without SI among veterans for whom data were available.

### 3.2. Sequenced psychotherapy and CPT / PE initiation

Tables 2 and 3 compare the rates of CPT and PE initiation between veterans who received sequenced psychotherapy (SPI or SPG) prior to CPT or PE and those who did not, stratified by race/ethnicity and risk group.

#### 3.2.1. Individual sequenced psychotherapy (SPI)

SPI was associated with increased initiation of CPT (range: 4.2 % to 6.7 %, p < .001) across all seven racial/ethnic groups except for Native Americans, and with increased initiation of PE among all groups except Asians, Native Americans and Pacific Islanders (p < .001); there were no racial/ethnic groups for which SPI was associated with decreased CPT or PE initiation. CPT and PE initiation rates were also significantly increased following SPI for veterans with heavy alcohol use (6.0 % and 2.1 %, respectively, p < .001) and CPT initiation rates were increased following SPI for those experiencing SI (3.5 %, p < .001); PE initiation rate for those with SI was not significant.

#### 3.2.2. Group sequenced psychotherapy (SPG)

SPG was associated with smaller increases than SPI in initiation of CPT for NHW veterans as well as all veterans in aggregate, while increases in PE initiation were small and significant only for NHW veterans. Among veterans with heavy alcohol use, SPG was associated with significant increases in CPT initiation for NHW (4.2 %) and BIPOC (2.2 %) veterans in the aggregate (p < .001), while for SPG there was no significant increases in PE initiation in any group. There were no significant reductions in CPT or PE initiation associated with SPI or SPG for any combination of race/ethnicity and risk group.

### 3.3. Sequenced psychotherapy and CPT / PE retention

Tables 4 and 5 detail CPT and PE retention rates by individual vs. group sequenced psychotherapy modality, race/ethnicity and risk group. Sequenced treatment was associated with increased retention in both CPT and PE, with the largest increases in treatment retention seen for PE following sequenced group treatment.

#### 3.3.1. Individual sequenced psychotherapy (SPI)

Among all veterans in aggregate, SPI was associated with significantly increased retention rates for CPT and PE: 8.0 % and 8.2 %, respectively (*p <* .001), compared to veterans receiving ≤ 1 session of SPI. Among BIPOC veterans, SPI was associated with greater increases in retention, both for CPT (8.9 %, *p <* .001) and for PE (9.1 %, *p <* .001); among Black veterans, the associated increase in CPT retention was also 8.9 %, compared to 7.6 % in NHW veterans ( < .001) and the increase in PE retention was not significant. Among veterans with heavy alcohol use, SPI was associated with a greater increase in retention in CPT, compared to all veterans in the aggregate (11.5 % vs. 8.0 %, respectively, p < .001). There was no significant change in the rate of retention in PE among veterans with heavy alcohol use, or in retention in CPT or PE in veterans with SI.

#### 3.3.2. Group sequenced psychotherapy (SPG)

Overall, SPG was associated with greater increases of retention in PE than in CPT. Among all veterans, SPG was associated with a 8.7 % increase of retention in PE and a 3.4 % increase of retention in CPT (*p <* .001). Among Black veterans, the increase in PE retention following SPG was 9.7 %, compared to 7.7 % in NHW veterans (*p* = .001). There was no demographic or risk group for which SPI or SPG was associated with a decrease in CPT or PE retention. For veterans with heavy alcohol use, SPG was associated with a greater increase in CPT retention compared to veterans in the aggregate (9.0 % vs. 3.4 %, p < .001), and the same was true for the increase in PE retention (11.5 % vs. 8.7 %, p < .001) following SPG, with the largest effects seen for BIPOC veterans (22.0 % increased PE retention, p < .001), Black veterans (13.1 % increased CPT retention, *p <* .001) and HW veterans (21.7 % increased CPT retention, *p <* .001). By comparison, there was a 7.3 % increase in CPT retention and no significant change in PE retention following SPG for NHW veterans with heavy drinking. Among veterans experiencing SI, SPG was not associated with significant changes in CPT or PE retention.

## 4. Discussion

To our knowledge, this is the first study to assess initiation of and retention in TF-EBPs using a comprehensive national sample of veterans seeking treatment for PTSD in VA outpatient settings. Given the staggering cost and burden of suffering involved, it remains a question of urgent concern how VA can best deploy finite psychotherapy resources to maximize the reach and effectiveness of evidence-based PTSD treatments. Our findings suggest that sequenced psychotherapy may improve CPT and PE retention rates without reducing initiation of these first-line PTSD treatments, particularly for BIPOC veterans and veterans with heavy drinking. Using national VA administrative data, we included all outpatient care settings to address limitations of studies based in PTSD specialty clinics and were powered to examine demographic and risk-defined subpopulations. Using a framework for identifying PTSD-focused episodes of care, we followed the trajectories of 490,097 veterans who presented for treatment over the 74-month study period, measuring initiation and retention in CPT and PE and assessing the impact of sequenced psychotherapy preceding CPT and PE on these variables. Importantly, this large dataset adds to our understanding of CPT and PE engagement across racial and ethnic groups, an area of urgent research need (Maguen et al., 2023; McClendon et al., 2020).

Our data support recent findings that TF-EBP uptake remains stubbornly low in VA (Cameron et al., 2023; Maguen et al., 2020); among veterans entering care for PTSD, only 9.5 % initiated CPT and 3.4 % initiated PE. It is unclear whether this low uptake primarily reflects veteran treatment preferences or whether it results from a failure of clinicians to offer CPT and PE. Importantly, however, our results suggest that sequenced individual and group psychotherapy preceding CPT and PE does not divert veterans from initiating these treatments, a key concern raised about sequenced or preparatory psychotherapy (Dedert et al., 2021; Wiedeman et al., 2020). There were no demographic groups in which sequenced individual or group therapies delivered before CPT or PE was associated with decreased initiation of these treatments; this observation also held for veterans with heavy drinking or SI in all demographic groups.

Our data is also consistent with prior findings that TF-EBP dropout among veterans remains persistently high (Cameron et al., 2023); of the veterans who initiated CPT or PE in our national sample, retention rates were only 46.4 % and 42.3 %, respectively. Retention rates were lower among BIPOC veterans. CPT and PE retention was also lower among veterans with heavy alcohol use, though there was no significant difference in treatment retention for veterans with SI. Importantly, our findings suggest that sequenced treatments may play a role in maintaining engagement in CPT and PE, particularly for BIPOC veterans and veterans with heavy alcohol use. Among veterans with heavy alcohol use, sequenced individual treatments were associated with a greater increase of retention in CPT, and the same held for sequenced group treatments and increases in both CPT and PE retention. For BIPOC veterans with heavy alcohol use, increases in CPT and PE retention were of greater magnitude, though were not significant for PE retention following SPI. Although it is unclear from our data what interventions were employed in SP sessions, it appears likely that treatments specifically focused on addressing substance use prior to initiating CPT or PE may provide particular benefit for veterans with heavy alcohol use.

Compared to NHW veterans, Black veterans had greater increases in CPT retention following SPI and in PE retention following SPG. Among Black veterans with heavy drinking, the increase in CPT retention following SPG was even greater compared to NHW veterans. Most strikingly, among HW veterans, those with heavy drinking who received SPG had a 21.7 % increase in CPT retention. This finding is particularly salient, given existing barriers to care in this population; published evidence shows that Hispanic and Latino veterans are less likely to receive pharmacotherapy and psychotherapy after a diagnosis of PTSD than other groups (Rosen et al., 2019; Spoont et al., 2015, 2017).

Our results suggest that a sequenced treatment approach to PTSD care may have a role to play in ensuring successful delivery of CPT and PE to BIPOC veterans and veterans with heavy drinking. In sequential or “phase-based” treatment, the initial phase of treatment provides a skills-based and relational foundation to support trauma processing (Cloitre, 2015; Cloitre et al., 2012; Courtois and Ford, 2013; Herman, 1992; ISTSS Guidelines Committee, 2019). This type of treatment can provide skills for addressing current stressors and newly experienced traumatic events, which may be of particular importance in minoritized groups given the chronic nature and cumulative effects of race-based stressors and traumatic events (Williams et al., 2018). For example, one study found that among veterans newly diagnosed with PTSD, African Americans were more likely to stay engaged in treatment when therapy focused on military stressors about half of the time (leaving room for focus on current concerns), while White veterans were most likely to remain in care when military stressors were the primary focus (M. Spoont et al., 2017).

Establishing safety in the therapeutic relationship is a key task of psychotherapy in the first phase of sequenced care. BIPOC veterans are more likely to feel misunderstood, uncared for, and unsafe at the VA, whether at the hands of providers, other veterans, or the system as a whole (Eliacin et al., 2020; Jones et al., 2000; Rickles et al., 2010; M. Spoont et al., 2017). The first phase of treatment may allow for more interactions and opportunities for BIPOC veterans to assess trustworthiness and care (M. Spoont et al., 2017) and to gain awareness of potential racial biases of providers and the diversity represented among staff (Eliacin et al., 2020). Further, this phase can provide a frame within which to assess the impact of inequity, covert or overt exposure to racism and discrimination, and race-based trauma on the lived experience of PTSD, which can then inform the course of TF-EBP (Maguen, Batten, et al., 2023; Williams et al., 2014). These affirming interactions, where identities and cultural differences in the therapeutic relationship are acknowledged and explored, provide a vital foundation for establishing trust and safety in addition to tailoring the course of care (Williams et al., 2014).

Our finding that sequenced group treatment may facilitate engagement of BIPOC veterans in CPT and PE—particularly those who have heavy alcohol use—must be viewed in the context of evidence suggesting a complex relationship of BIPOC veterans to group treatment in VA. BIPOC veterans with PTSD are referred to group treatment more frequently than NHW veterans (Mott et al., 2014; M. R. Spoont et al., 2017), with Black veterans disproportionately represented in group CPT (Maguen, Batten, et al., 2023). This raises important concerns, as research suggests that group treatments for PTSD may be less efficacious than individual therapy (Resick et al., 2016; Sloan et al., 2012). Although work has been done by Spoont and colleagues (M. R. Spoont et al., 2017) to examine drivers of racial and ethnic variation in receiving individual psychotherapy, it is unclear to what extent these overall disparities may be driven by under-resourced health care systems, provider bias, structural racism in the VA, veteran preference and values, or attempts of the referring provider to culturally tailor a veteran’s care trajectory.

Keeping these important concerns in mind, it is also worth considering that sequenced group treatment may offer particular benefits for racially and ethnically minoritized veterans. Groups in which BIPOC veterans are in community may create a sense of safety and provide a forum for sharing concerns and collective trauma experiences not addressed in other VA spaces (Jones et al., 2000; C. Wang et al., 2023); these factors have been noted as foundational in a novel group intervention addressing race-based stress within the VA (Carlson et al., 2018). The sense of safety gained through group treatment may serve as a facilitator of trust and openness to engage in TF-EBP. Sequenced group treatment may provide more culturally-aligned opportunities for trauma recovery than manualized TF-EBP interventions, which were not developed to address the experiences of race-based or societal/ethnic traumas or their intersection with other traumatic events (Bryant-Davis, 2019; Carlson et al., 2018). Importantly, there is evidence to suggest that BIPOC veterans experience less improvement in PTSD symptoms after TF-EBP compared to White veterans (Grau et al., 2022; Lamp et al., 2019; Maguen, Batten, et al., 2023).

A key limitation of this study is that it utilizes CPT and PE completion as an outcome measure, lacking direct measures of symptom improvement. For BIPOC veterans, TF-EBP completion may be insufficient, and at worst, problematic as an indicator of treatment success. The failure of current manualized TF-EBPs to address race-based or societal/ethnic traumas may lead providers to overlook significant traumatic experiences when working with racial and ethnic minoritized veterans. Indeed, there is evidence that engagement in PTSD care can widen pretreatment health disparities; a recent study by Spoont and colleagues (M. Spoont et al., 2021) found that among veterans who initiated PTSD psychotherapy and pharmacotherapy, White veterans were more likely to experience improvements in PTSD symptoms when compared to Latinx and African American veterans. Thus, our finding that sequenced individual and group treatment is associated with greater increases in CPT and PE retention for some groups of BIPOC veterans than for NHW veterans does not equate with improved clinical outcomes.

Another limitation of this research is that we relied on health factors generated by CPT and PE note templates to indicate sessions of TF-EBP and treated all other sessions as sequenced psychotherapy. This approach did not identify therapeutic modalities employed in SP sessions, leaving their content unspecified; thus, it is not possible to identify to what extent a skills-oriented or phase-based approach was used prior to initiating CPT or PE, or whether SP sessions were used to address specific concerns of BIPOC veterans or the impact of race-based trauma. Likewise, by relying on CPT and PE note templates, we excluded other TF-EBPs from the analysis, such as Eye Movement Desensitization and Reprocessing, Written Exposure Therapy, CPT in group format, and other first-line PTSD psychotherapies, sessions of which would have been incorrectly designated as SP. In addition, this study relied on administrative data for race and ethnicity, which is subject to misclassification (Onoye et al., 2017), and we did not examine the intersection of identity variables. Further, effects of race and ethnicity may be confounded by socioeconomic factors, neighborhood characteristics, and other social determinants of health, many of which are not measured in administrative data. Since PHQ9 data from the prior year was only available for 23 % of the sample, the generalizability of our findings with respect to SI may be limited, as there may have been systematic differences in the group to whom PHQ9 measures were administered by clinicians, such as increased concern for depression. (In contrast, AUDIT-C data from the prior 18 months were available for 85 % of the sample and were generated via a scheduled clinical reminder, making systematic bias less likely in the group for whom data about heavy vs. light drinking were available.) Finally, this naturalistic, observational approach introduced potential bias by neglecting to control for factors that might influence the selection of patients for sequenced psychotherapy.

Further research is needed to identify specific characteristics of sequenced psychotherapy that are associated with improved TF-EBP initiation and retention; while we have described in previous work a successful, specialty clinic-based model for sequential therapy which includes a cognitive behavioral, skills-based stabilization component (Staudenmeyer et al., 2022), larger studies are needed. Likewise, future research should aim to improve the accuracy of race and ethnicity variables drawn from health records. Innovations in natural language processing, such as those developed by Maguen and colleagues (Maguen et al., 2018; Maguen, Madden, et al., 2023), could be applied at scale to identify the full range of TF-EBPs in the medical record, characterize components of effective sequential therapies and extract clinical outcome measures. Crucially, ongoing research into novel evidence-based PTSD therapies must continue, so that an expanding menu of effective and culturally-affirming treatments can be brought within reach of all veterans who carry the daily burdens of trauma-related suffering and disability.

## Figures and Tables

**Table 1:** Dissemination of CPT and PE among veterans entering mental health treatment between 10/1/14 and 11/30/20 who had 3 encounters in which PTSD was designated the primary diagnosis. The first section compares initiation and dropout rates of BIPOC veterans to those of Non-Hispanic White veterans. The second and third sections compare rates of veterans with the identified risk factor to those in the same demographic category without the corresponding risk factor. (Significance: p < 0.001).

			CPT/PE Dissemination (Oct 2014 - Nov 2020) (n=490,097)																							
			Initiation												Retention											
			CPT						PE						CPT						PE					
	n		Race/Ethnicity		Non-Hisp. White		Δ%	p	Race/Ethnicity		Non-Hisp. White		Δ%	p	Race/Ethnicity		Non-Hisp. White		Δ%	p	Race/Ethnicity		Non-Hisp. White		Δ%	p
All Veterans	490,097		46,411	(9.5%)	-	-	-	-	16,556	(3.4%)	-	-	-	-	21,550	(46.4%)	-	-	-	-	6,998	(42.3%)	-	-	-	-
Non-Hispanic White	274,463		26,405	(9.6%)	-	-	-	-	9,084	(3.3%)	-	-	-	-	12,656	(47.9%)	-	-	-	-	3,921	(43.2%)	-	-	-	-
BIPOC	175,802	274,463	16,252	(9.2%)	26,405	(9.6%)	−0.4% *	<.001	6,107	(3.5%)	9,084	(3.3%)	+0.2%	.003	7,120	(43.8%)	12,656	(47.9%)	+4.1% *	<.001	2,510	(41.1%)	3,921	(43.2%)	+2.1%	.01
Black	114,744	274,463	10,483	(9.1%)	26,405	(9.6%)	−0.5% *	<.001	3,890	(3.4%)	9,084	(3.3%)	+0.1%	.20	4,593	(43.8%)	12,656	(47.9%)	+4.1% *	<.001	1,606	(41.3%)	3,921	(43.2%)	+1.9%	.05
Hispanic White	35,022	274,463	3,415	(9.8%)	26,405	(9.6%)	+0.1%	.44	1,322	(3.8%)	9,084	(3.3%)	+0.5% *	<.001	1,497	(43.8%)	12,656	(47.9%)	+4.1% *	<.001	503	(38.0%)	3,921	(43.2%)	+5.1% *	<.001
Asian	7,480	274,463	680	(9.1%)	26,405	(9.6%)	−0.5%	.13	264	(3.5%)	9,084	(3.3%)	+0.2%	.30	353	(51.9%)	12,656	(47.9%)	−4.0%	.04	119	(45.1%)	3,921	(43.2%)	−1.9%	.54
Native American	6,034	274,463	562	(9.3%)	26,405	(9.6%)	−0.3%	.42	201	(3.3%)	9,084	(3.3%)	+0.0%	.93	245	(43.6%)	12,656	(47.9%)	+4.3%	.04	92	(45.8%)	3,921	(43.2%)	−2.6%	.46
Pacific Islander	5,954	274,463	496	(8.3%)	26,405	(9.6%)	−1.3% *	<.001	205	(3.4%)	9,084	(3.3%)	+0.1%	.57	185	(37.3%)	12,656	(47.9%)	+10.6% *	<.001	95	(46.3%)	3,921	(43.2%)	−3.2%	.36
Multiracial	6,568	274,463	616	(9.4%)	26,405	(9.6%)	−0.2%	.51	225	(3.4%)	9,084	(3.3%)	+0.1%	.60	247	(40.1%)	12,656	(47.9%)	+7.8% *	<.001	95	(42.2%)	3,921	(43.2%)	+0.9%	.78
Missing Race/Ethn	39,832	274,463	3,754	(9.4%)	26,405	(9.6%)	−0.2%	.21	1,365	(3.4%)	9,084	(3.3%)	+0.1%	.22	1,774	(47.3%)	12,656	(47.9%)	+0.7%	.44	567	(41.5%)	3,921	(43.2%)	+1.6%	.26
Heavy Alcohol Use	Heavy Drinker	Light Drinker	Heavy Drinker		Light Drinker		Δ%	p	Heavy Drinker		Light Drinker		Δ%	p	Heavy Drinker		Light Drinker		Δ%	p	Heavy Drinker		Light Drinker		Δ%	p
All Veterans	67,885	298,025	6,704	(9.9%)	27,321	(9.2%)	+0.7% *	<.001	2,408	(3.5%)	9,838	(3.3%)	+0.2%	.001	2,941	(43.9%)	12,825	(46.9%)	+3.1% *	<.001	945	(39.2%)	4,257	(43.3%)	+4.0% *	<.001
Non-Hispanic White	38,039	166,375	3,906	(10.3%)	15,245	(9.2%)	+1.1% *	<.001	1,330	(3.5%)	5,335	(3.2%)	+0.3%	.004	1,763	(45.1%)	7,375	(48.4%)	+3.2% *	<.001	544	(40.9%)	2,369	(44.4%)	+3.5%	.02
BIPOC	24,071	108,841	2,225	(9.2%)	10,018	(9.2%)	+0.0%	.85	868	(3.6%)	3,768	(3.5%)	+0.1%	.27	932	(41.9%)	4,461	(44.5%)	+2.6%	.02	319	(36.8%)	1,589	(42.2%)	+5.4%	.003
Black	14,540	72,717	1,340	(9.2%)	6,630	(9.1%)	+0.1%	.71	490	(3.4%)	2,498	(3.4%)	−0.1%	.69	566	(42.2%)	2,934	(44.3%)	+2.0%	.18	196	(40.0%)	1,051	(42.1%)	+2.1%	.39
Hispanic White	5,914	20,419	538	(9.1%)	2,010	(9.8%)	−0.7%	.09	251	(4.2%)	729	(3.6%)	+0.7%	.02	227	(42.2%)	908	(45.2%)	+3.0%	.22	76	(30.3%)	296	(40.6%)	+10.3%	.004
Asian	929	4,693	83	(8.9%)	415	(8.8%)	+0.1%	.93	35	(3.8%)	155	(3.3%)	+0.5%	.47	48	(57.8%)	221	(53.3%)	−4.6%	.44	12	(34.3%)	74	(47.7%)	+13.5%	.15
Native American	889	3,603	90	(10.1%)	329	(9.1%)	+1.0%	.36	29	(3.3%)	127	(3.5%)	−0.3%	.70	39	(43.3%)	146	(44.4%)	+1.0%	.86	12	(41.4%)	60	(47.2%)	+5.9%	.57
Pacific Islander	922	3,499	88	(9.5%)	276	(7.9%)	+1.7%	.10	27	(2.9%)	124	(3.5%)	−0.6%	.36	21	(23.9%)	110	(39.9%)	+16.0%	.006	10	(37.0%)	54	(43.5%)	+6.5%	.53
Multiracial	877	3,910	86	(9.8%)	358	(9.2%)	+0.7%	.55	36	(4.1%)	135	(3.5%)	+0.7%	.35	31	(36.0%)	142	(39.7%)	+3.6%	.54	13	(36.1%)	54	(40.0%)	+3.9%	.67
Missing Race/Ethn	5,775	22,809	573	(9.9%)	2,058	(9.0%)	+0.9%	.03	210	(3.6%)	735	(3.2%)	+0.4%	.12	246	(42.9%)	989	(48.1%)	+5.1%	.03	82	(39.0%)	299	(40.7%)	+1.6%	.67
Suicidal Ideation	SI	No SI	SI		No SI		Δ%	p	SI		No SI		Δ%	p	SI		No SI		Δ%	p	SI		No SI		Δ%	p
All Veterans	11,838	81,087	1,513	(12.8%)	9,494	(11.7%)	+1.1% *	<.001	493	(4.2%)	3,684	(4.5%)	−0.4%	.06	679	(44.9%)	4,612	(48.6%)	+3.7%	.007	209	(42.4%)	1,600	(43.4%)	+1.0%	.66
Non-Hispanic White	6,032	44,173	799	(13.2%)	5,273	(11.9%)	+1.3%	.003	258	(0.0%)	1,968	(4.5%)	−4.5%	.53	365	(45.7%)	2,674	(50.7%)	+5.0%	.008	112	(43.4%)	910	(46.2%)	+2.8%	.39
BIPOC	4,852	30,816	593	(12.2%)	3,510	(11.4%)	+0.8%	.09	195	(4.0%)	1,462	(4.7%)	−0.7%	.03	252	(42.5%)	1,599	(45.6%)	+3.1%	.17	79	(40.5%)	589	(40.3%)	−0.2%	.95
Black	3,036	21,160	371	(12.2%)	2,383	(11.3%)	+1.0%	.12	117	(3.9%)	970	(4.6%)	−0.7%	.07	153	(41.2%)	1,076	(45.2%)	+3.9%	.16	52	(44.4%)	381	(39.3%)	−5.2%	.28
Hispanic White	1,004	5,878	122	(12.2%)	741	(12.6%)	−0.5%	.69	48	(4.8%)	294	(5.0%)	−0.2%	.77	54	(44.3%)	339	(45.7%)	+1.5%	.76	17	(35.4%)	122	(41.5%)	+6.1%	.43
Asian	271	1,066	31	(11.4%)	114	(10.7%)	+0.7%	.72	12	(4.4%)	60	(5.6%)	−1.2%	.43	16	(51.6%)	62	(54.4%)	+2.8%	.78	3	(25.0%)	27	(45.0%)	+20.0%	.20
Native American	160	830	20	(12.5%)	83	(10.0%)	+2.5%	.34	9	(5.6%)	38	(4.6%)	+1.0%	.57	14	(70.0%)	39	(47.0%)	−23.0%	.06	2	(22.2%)	20	(52.6%)	+30.4%	.10
Pacific Islander	211	808	31	(14.7%)	84	(10.4%)	+4.3%	.08	4	(1.9%)	52	(6.4%)	−4.5%	.010	7	(22.6%)	35	(41.7%)	+19.1%	.06	1	(25.0%)	22	(42.3%)	+17.3%	.50
Multiracial	170	1,074	18	(10.6%)	105	(9.8%)	+0.8%	.74	5	(2.9%)	48	(4.5%)	−1.5%	.36	8	(44.4%)	48	(45.7%)	+1.3%	.92	4	(80.0%)	17	(35.4%)	−44.6%	.05
Missing Race/Ethn	954	6,098	121	(12.7%)	711	(11.7%)	+1.0%	.36	40	(4.2%)	254	(4.2%)	+0.0%	.97	62	(51.2%)	339	(47.7%)	−3.6%	.47	18	(45.0%)	101	(39.8%)	−5.2%	.53

**Table 2:** Comparison of CPT and PE initiation rates for veterans who received ≥ 8 sessions of individual sequenced psychotherapy with those who received ≤ 1 session of individual sequenced treatment. (Significance: p <0.001).

		Individual Sequenced Psychotherapy (SPI): Initiation of CPT/PE													
		≤ 1 session					≥ 8 sessions					Increase in Initiation			
	n	n	CPT Initiation		PE Initiation		n	CPT Initiation		PE Initiation		CPT	p value	PE	p value
All Veterans	490,097	158,100	11,143	(7.0%)	3,951	(2.5%)	48,607	5,647	(11.6%)	2,006	(4.1%)	4.6%*	<.001	1.6%*	<.001
non-Hispanic White	274,463	87,116	6,493	(7.5%)	2,149	(2.5%)	28,531	3,337	(11.7%)	1,124	(3.9%)	4.2%*	<.001	1.5%*	<.001
BIPOC	175,802	57,883	3,718	(6.4%)	1,471	(2.5%)	16,078	1,842	(11.5%)	716	(4.5%)	5.0%*	<.001	1.9%*	<.001
Black	114,744	38,345	2,408	(6.3%)	972	(2.5%)	10,106	1,154	(11.4%)	452	(4.5%)	5.1%*	<.001	1.9%*	<.001
Hispanic White	35,022	11,132	773	(6.9%)	295	(2.7%)	3,238	388	(12.0%)	144	(4.4%)	5.0%*	<.001	1.8%*	<.001
Asian	7,480	2,542	143	(5.6%)	58	(2.3%)	788	97	(12.3%)	32	(4.1%)	6.7%*	<.001	1.8%	.007
Native American	6,034	1,948	145	(7.4%)	54	(2.8%)	672	62	(9.2%)	26	(3.9%)	1.8%	.14	1.1%	.15
Pacific Islander	5,954	1,910	114	(6.0%)	47	(2.5%)	567	59	(10.4%)	24	(4.2%)	4.4%*	<.001	1.8%	.03
Multiracial	6,568	2,006	135	(6.7%)	45	(2.2%)	707	82	(11.6%)	38	(5.4%)	4.9%*	<.001	3.1%*	<.001
Missing Race/Ethn	39,832	13,101	932	(7.1%)	331	(2.5%)	3,998	468	(11.7%)	166	(4.2%)	4.6%*	<.001	1.6%*	<.001
Heavy Alcohol Use	67,885	20,638	1,431	(6.9%)	526	(2.5%)	8,081	1,049	(13.0%)	375	(4.6%)	6.0%*	<.001	2.1%*	<.001
Non-Hispanic White	38,039	11,332	848	(7.5%)	293	(2.6%)	4,868	658	(13.5%)	202	(4.1%)	6.0%*	<.001	1.6%*	<.001
BIPOC	24,071	7,424	434	(5.8%)	197	(2.7%)	2,589	308	(11.9%)	140	(5.4%)	6.1%*	<.001	2.8%*	<.001
Black	14,540	4,446	242	(5.4%)	113	(2.5%)	1,577	193	(12.2%)	89	(5.6%)	6.8%*	<.001	3.1%*	<.001
Hispanic White	5,914	1,868	116	(6.2%)	51	(2.7%)	575	69	(12.0%)	39	(6.8%)	5.8%*	<.001	4.1%*	<.001
Asian	929	303	15	(5.0%)	7	(2.3%)	100	13	(13.0%)	3	(3.0%)	8.0%	.006	0.7%	.70
Native American	889	283	22	(7.8%)	7	(2.5%)	123	16	(13.0%)	3	(2.4%)	5.2%	.10	−0.0%	.98
Pacific Islander	922	273	19	(7.0%)	8	(2.9%)	107	7	(6.5%)	3	(2.8%)	−0.4%	.88	−0.1%	.95
Multiracial	877	251	20	(8.0%)	11	(4.4%)	107	10	(9.3%)	3	(2.8%)	1.4%	.67	−1.6%	.48
Missing Race/Ethn	1,355	462	41	(8.9%)	9	(1.9%)	137	16	(11.7%)	10	(7.3%)	2.8%	.33	5.4%	.002
Suicidal Ideation	11,838	2,794	279	(10.0%)	93	(3.3%)	1,692	228	(13.5%)	88	(5.2%)	3.5%*	<.001	1.9%	.002
Non-Hispanic White	6,032	1,374	149	(10.8%)	45	(3.3%)	921	131	(14.2%)	40	(4.3%)	3.4%	.02	1.1%	.18
BIPOC	4,852	1,165	106	(9.1%)	40	(3.4%)	647	80	(12.4%)	36	(5.6%)	3.3%	.03	2.1%	.03
Black	3,036	735	67	(9.1%)	25	(3.4%)	403	56	(13.9%)	19	(4.7%)	4.8%	.01	1.3%	.27
Hispanic White	1,004	234	22	(9.4%)	8	(3.4%)	117	12	(10.3%)	10	(8.5%)	0.9%	.80	5.1%	.04
Asian	271	71	4	(5.6%)	1	(1.4%)	45	3	(6.7%)	3	(6.7%)	1.0%	.82	5.3%	.13
Native American	160	42	3	(7.1%)	4	(9.5%)	26	2	(7.7%)	1	(3.8%)	0.5%	.93	−5.7%	.38
Pacific Islander	211	48	7	(14.6%)	2	(4.2%)	31	3	(9.7%)	0	(0.0%)	−4.9%	.52	−4.2%	.25
Multiracial	170	35	3	(8.6%)	0	(0.0%)	25	4	(16.0%)	3	(12.0%)	7.4%	.38	12.0%	.04
Missing Race/Ethn	954	255	24	(9.4%)	8	(3.1%)	124	17	(13.7%)	12	(9.7%)	4.3%	.21	6.5%	.008

**Table 3:** Comparison of CPT and PE initiation rates for veterans who received ≥ 8 sessions of group sequenced psychotherapy with those who received ≤1 session of group sequenced treatment. (Significance: p <0.001).

		Group Sequenced Psychotherapy (SPG): Initiation of CPT/PE													
		≤ 1 session					≥ 8 sessions					Increase in Initiation			
	n	n	CPT Initiation		PE Initiation		n	CPT Initiation		PE Initiation		CPT	p value	PE	p value
All Veterans	490,097	392,864	36,458	(9.3%)	13,181	(3.4%)	44,960	4,729	(10.5%)	1,588	(3.5%)	1.2%*	<.001	0.2%	.05
Non-Hispanic White	274,463	226,342	21,144	(9.3%)	7,383	(3.3%)	23,447	2,666	(11.4%)	870	(3.7%)	2.0%*	<.001	0.4%*	<.001
BIPOC	175,802	134,384	12,318	(9.2%)	4,710	(3.5%)	18,097	1,726	(9.5%)	596	(3.3%)	0.4%	.10	−0.2%	.14
Black	114,744	86,143	7,790	(9.0%)	2,949	(3.4%)	12,424	1,167	(9.4%)	395	(3.2%)	0.4%	.20	−0.2%	.16
Hispanic White	35,022	27,593	2,685	(9.7%)	1,054	(3.8%)	3,118	306	(9.8%)	112	(3.6%)	0.1%	.88	−0.2%	.53
Asian	7,480	5,958	550	(9.2%)	227	(3.8%)	699	63	(9.0%)	16	(2.3%)	−0.2%	.85	−1.5%	.04
Native American	6,034	4,736	427	(9.0%)	148	(3.1%)	685	72	(10.5%)	27	(3.9%)	1.5%	.21	0.8%	.26
Pacific Islander	5,954	4,743	392	(8.3%)	161	(3.4%)	559	46	(8.2%)	26	(4.7%)	−0.0%	.98	1.3%	.13
Multiracial	6,568	5,211	474	(9.1%)	171	(3.3%)	612	72	(11.8%)	20	(3.3%)	2.7%	.03	−0.0%	.99
Missing Race/Ethn	39,832	32,138	2,996	(9.3%)	1,088	(3.4%)	3,416	337	(9.9%)	122	(3.6%)	0.5%	.30	0.2%	.57
Heavy Alcohol Use	67,885	51,915	4,849	(9.3%)	1,835	(3.5%)	8,421	1,081	(12.8%)	310	(3.7%)	3.5%*	<.001	0.1%	.50
Non-Hispanic White	38,039	29,730	2,851	(9.6%)	1,016	(3.4%)	4,687	647	(13.8%)	190	(4.1%)	4.2%*	<.001	0.6%	.03
BIPOC	24,071	17,724	1,578	(8.9%)	656	(3.7%)	3,096	345	(11.1%)	96	(3.1%)	2.2%*	<.001	−0.6%	.10
Black	14,540	10,410	917	(8.8%)	354	(3.4%)	2,014	223	(11.1%)	68	(3.4%)	2.3%	.001	−0.0%	.96
Hispanic White	5,914	4,579	405	(8.8%)	204	(4.5%)	622	70	(11.3%)	18	(2.9%)	2.4%	.05	−1.6%	.07
Asian	929	708	64	(9.0%)	30	(4.2%)	110	12	(10.9%)	1	(0.9%)	1.9%	.53	−3.3%	.09
Native American	889	647	59	(9.1%)	16	(2.5%)	146	18	(12.3%)	6	(4.1%)	3.2%	.24	1.6%	.28
Pacific Islander	922	727	69	(9.5%)	22	(3.0%)	90	11	(12.2%)	3	(3.3%)	2.7%	.41	0.3%	.87
Multiracial	877	653	64	(9.8%)	30	(4.6%)	114	11	(9.6%)	0	(0.0%)	−0.2%	.96	−4.6%	.02
Missing Race/Ethn	1,355	1,108	118	(10.6%)	45	(4.1%)	101	5	(5.0%)	3	(3.0%)	−5.7%	.07	−1.1%	.59
Suicidal Ideation	11,838	8,795	1,129	(12.8%)	380	(4.3%)	1,444	183	(12.7%)	62	(4.3%)	−0.2%	.86	−0.0%	.96
Non-Hispanic White	6,032	4,628	619	(13.4%)	211	(4.6%)	718	100	(13.9%)	26	(3.6%)	0.6%	.69	−0.9%	.26
BIPOC	4,852	3,427	414	(12.1%)	137	(4.0%)	613	73	(11.9%)	31	(5.1%)	−0.2%	.90	1.1%	.23
Black	3,036	2,104	244	(11.6%)	81	(3.8%)	392	52	(13.3%)	25	(6.4%)	1.7%	.35	2.5%	.02
Hispanic White	1,004	741	100	(13.5%)	31	(4.2%)	108	7	(6.5%)	6	(5.6%)	−7.0%	.04	1.4%	.51
Asian	271	196	26	(13.3%)	11	(5.6%)	41	5	(12.2%)	0	(0.0%)	−1.1%	.85	−5.6%	.12
Native American	160	120	12	(10.0%)	8	(6.7%)	19	4	(21.1%)	0	(0.0%)	11.1%	.16	−6.7%	.25
Pacific Islander	211	153	20	(13.1%)	4	(2.6%)	31	1	(3.2%)	0	(0.0%)	−9.8%	.12	−2.6%	.36
Multiracial	170	113	12	(10.6%)	2	(1.8%)	22	4	(18.2%)	0	(0.0%)	7.6%	.32	−1.8%	.53
Missing Race/Ethn	954	740	96	(13.0%)	32	(4.3%)	113	10	(8.8%)	5	(4.4%)	−4.1%	.22	0.1%	.96

**Table 4:** Comparison of CPT and PE retention rates for veterans who received ≥ 8 sessions of individual sequenced psychotherapy with those who received ≤ 1 session of individual sequenced treatment. (Significance: p <0.001).

	Individual Sequenced Psychotherapy (SPI): Retention in CPT/PE															
	≤ 1 session						≥ 8 sessions						Increase in Retention			
	CRT			PE			CRT			PE			CPT		PE	
	n	Retention		n	Retention		n	Retention		n	Retention		%	p value	%	p value
All Veterans	11,143	4,890	(43.9%)	3,951	1,533	(38.8%)	5,647	2,930	(51.9%)	2,006	943	(47.0%)	8.0%*	<.001	8.2%*	<.001
Non-Hispanic White	6,493	2,953	(45.5%)	2,149	859	(40.0%)	3,337	1,771	(53.1%)	1,124	530	(47.2%)	7.6%*	<.001	7.2%*	<.001
BIPOC	3,718	1,515	(40.7%)	1,471	548	(37.3%)	1,842	915	(49.7%)	716	332	(46.4%)	8.9%*	<.001	9.1%*	<.001
Black	2,408	971	(40.3%)	972	361	(37.1%)	1,154	568	(49.2%)	452	203	(44.9%)	8.9%*	<.001	7.8%	.005
Hispanic White	773	321	(41.5%)	295	99	(33.6%)	388	190	(49.0%)	144	70	(48.6%)	7.4%	.02	15.1%	.002
Asian	143	79	(55.2%)	58	29	(50.0%)	97	61	(62.9%)	32	14	(43.8%)	7.6%	.24	−6.3%	.57
Native American	145	60	(41.4%)	54	25	(46.3%)	62	27	(43.5%)	26	12	(46.2%)	2.2%	.77	−0.1%	.99
Pacific Islander	114	41	(36.0%)	47	15	(31.9%)	59	25	(42.4%)	24	14	(58.3%)	6.4%	.41	26.4%	.03
Multiracial	135	43	(31.9%)	45	19	(42.2%)	82	44	(53.7%)	38	19	(50.0%)	21.8%	.001	7.8%	.48
Missing Race/Ethn	932	422	(45.3%)	331	126	(38.1%)	468	244	(52.1%)	166	81	(48.8%)	6.9%	.02	10.7%	.02
Heavy Alcohol Use	1,431	555	(38.8%)	526	192	(36.5%)	1,049	528	(50.3%)	375	177	(47.2%)	11.5%*	<.001	10.7%	.001
Non-Hispanic White	848	346	(40.8%)	293	116	(39.6%)	658	329	(50.0%)	202	97	(48.0%)	9.2%*	<.001	8.4%	.06
BIPOC	434	161	(37.1%)	197	67	(34.0%)	308	161	(52.3%)	140	64	(45.7%)	15.2%*	<.001	11.7%	.03
Black	242	88	(36.4%)	113	41	(36.3%)	193	96	(49.7%)	89	38	(42.7%)	13.4%	.005	6.4%	.35
Hispanic White	116	49	(42.2%)	51	14	(27.5%)	69	41	(59.4%)	39	21	(53.8%)	17.2%	.02	26.4%	.01
Asian	15	7	(46.7%)	7	2	(28.6%)	13	9	(69.2%)	3	1	(33.3%)	22.6%	.23	4.8%	.88
Native American	22	7	(31.8%)	7	3	(42.9%)	16	8	(50.0%)	3	1	(33.3%)	18.2%	.26	−9.5%	.78
Pacific Islander	19	4	(21.1%)	8	1	(12.5%)	7	-	(0.0%)	3	2	(66.7%)	−21.1%	.19	54.2%	.07
Multiracial	20	6	(30.0%)	11	6	(54.5%)	10	7	(70.0%)	3	1	(33.3%)	40.0%	.04	−21.2%	.51
Missing Race/Ethn	41	17	(41.5%)	9	2	(22.2%)	16	9	(56.3%)	10	6	(60.0%)	14.8%	.31	37.8%	.10
Suicidal Ideation	279	105	(37.6%)	93	41	(44.1%)	228	115	(50.4%)	88	37	(42.0%)	12.8%	.004	−2.0%	.78
Non-Hispanic White	149	62	(41.6%)	45	22	(48.9%)	131	69	(52.7%)	40	15	(37.5%)	11.1%	.06	−11.4%	.29
BIPOC	106	33	(31.1%)	40	18	(45.0%)	80	38	(47.5%)	36	16	(44.4%)	16.4%	.02	−0.6%	.96
Black	67	18	(26.9%)	25	14	(56.0%)	56	26	(46.4%)	19	7	(36.8%)	19.6%	.02	−19.2%	.21
Hispanic White	22	9	(40.9%)	8	3	(37.5%)	12	4	(33.3%)	10	4	(40.0%)	−7.6%	.66	2.5%	.91
Asian	4	2	(50.0%)	1	1	(100%)	3	2	(66.7%)	3	1	(33.3%)	16.7%	.66	−66.7%	.25
Native American	3	2	(66.7%)	4	0	(0.0%)	2	2	(100%)	1	1	(100%)	33.3%	.36	100%	.03
Pacific Islander	7	2	(28.6%)	2	0	(0.0%)	3	1	(33.3%)	0	-	-	4.8%	.88	-	-
Multiracial	3	-	(0.0%)	0	-	-	4	3	(75.0%)	3	3	(100%)	75.0%	.05	-	-
Missing Race/Ethn	24	10	(41.7%)	8	1	(12.5%)	17	8	(47.1%)	12	6	(50.0%)	5.4%	.73	37.5%	.08

**Table 5:** Comparison of CPT and PE retention rates for veterans who received ≥ 8 sessions of group sequenced psychotherapy with those who received ≤ 1 session of group sequenced treatment. (Significance: p <0.001).

	Group Sequenced Psychotherapy (SPG): Retention in CPT/PE															
	≤ 1 session						≥ 8 sessions						Increase in Retention			
	CRT			PE			CRT			PE			CPT		PE	
	n	Retention		n	Retention		n	Retention		n	Retention		%	p value	%	p value
All Veterans	36,458	16,689	(45.8%)	13,181	5,380	(40.8%)	4,729	2,325	(49.2%)	1,588	786	(49.5%)	3.4% *	<.001	8.7% *	<.001
Non-Hispanic White	21,144	9,964	(47.1%)	7,383	3,065	(41.5%)	2,666	1,353	(50.8%)	870	428	(49.2%)	3.6% *	<.001	7.7% *	<.001
BIPOC	12,318	5,319	(43.2%)	4,710	1,885	(40.0%)	1,726	815	(47.2%)	596	296	(49.7%)	4.0%	.002	9.6% *	<.001
Black	7,790	3,371	(43.3%)	2,949	1,191	(40.4%)	1,167	562	(48.2%)	395	198	(50.1%)	4.9%	.002	9.7% *	<.001
Hispanic White	2,685	1,138	(42.4%)	1,054	383	(36.3%)	306	156	(51.0%)	112	52	(46.4%)	8.6%	.004	10.1%	.04
Asian	550	287	(52.2%)	227	99	(43.6%)	63	33	(52.4%)	16	9	(56.3%)	0.2%	.98	12.6%	.33
Native American	427	193	(45.2%)	148	71	(48.0%)	72	23	(31.9%)	27	15	(55.6%)	−13.3%	.04	7.6%	.47
Pacific Islander	392	151	(38.5%)	161	68	(42.2%)	46	10	(21.7%)	26	15	(57.7%)	−16.8%	.03	15.5%	.14
Multiracial	474	179	(37.8%)	171	73	(42.7%)	72	31	(43.1%)	20	7	(35.0%)	5.3%	.39	−7.7%	.51
Missing Race/Ethn	2,996	1,406	(46.9%)	1,088	430	(39.5%)	337	157	(46.6%)	122	62	(50.8%)	−0.3%	.91	11.3%	.02
Heavy Alcohol Use	4,849	2,031	(41.9%)	1,835	683	(37.2%)	1,081	550	(50.9%)	310	151	(48.7%)	9.0% *	<.001	11.5% *	<.001
Non-Hispanic White	2,851	1,227	(43.0%)	1,016	398	(39.2%)	647	326	(50.4%)	190	86	(45.3%)	7.3% *	<.001	6.1%	.12
BIPOC	1,578	627	(39.7%)	656	225	(34.3%)	345	182	(52.8%)	96	54	(56.3%)	13.0%*	<.001	22.0% *	<.001
Black	917	365	(39.8%)	354	131	(37.0%)	223	118	(52.9%)	68	38	(55.9%)	13.1%*	<.001	18.9%	.004
Hispanic White	405	161	(39.8%)	204	58	(28.4%)	70	43	(61.4%)	18	10	(55.6%)	21.7% *	<.001	27.1%	.02
Asian	64	34	(53.1%)	30	10	(33.3%)	12	8	(66.7%)	1	0	(0.0%)	13.5%	.39	−33.3%	.48
Native American	59	29	(49.2%)	16	8	(50.0%)	18	6	(33.3%)	6	3	(50.0%)	−15.8%	.24	0.0%	1
Pacific Islander	69	17	(24.6%)	22	6	(27.3%)	11	1	(9.1%)	3	3	(100%)	−15.5%	.25	72.7%	.01
Multiracial	64	21	(32.8%)	30	12	(40.0%)	11	6	(54.5%)	0	-	-	21.7%	.17	-	-
Missing Race/Ethn	118	55	(46.6%)	45	18	(40.0%)	5	2	(40.0%)	3	2	(66.7%)	−6.6%	.77	26.7%	.36
Suicidal Ideation	1,129	507	(44.9%)	380	161	(42.4%)	183	86	(47.0%)	62	25	(40.3%)	2.1%	.60	−2.0%	.76
Non-Hispanic White	619	282	(45.6%)	211	89	(42.2%)	100	46	(46.0%)	26	12	(46.2%)	0.4%	.93	4.0%	.70
BIPOC	414	174	(42.0%)	137	58	(42.3%)	73	36	(49.3%)	31	11	(35.5%)	7.3%	.25	−6.9%	.48
Black	244	98	(40.2%)	81	38	(46.9%)	52	24	(46.2%)	25	10	(40.0%)	6.0%	.43	−6.9%	.54
Hispanic White	100	45	(45.0%)	31	13	(41.9%)	7	4	(57.1%)	6	1	(16.7%)	12.1%	.53	−25.3%	.24
Asian	26	12	(46.2%)	11	3	(27.3%)	5	4	(80.0%)	0	-	-	33.8%	.17	-	-
Native American	12	9	(75.0%)	8	2	(25.0%)	4	2	(50.0%)	0	-	-	−25.0%	.35	-	-
Pacific Islander	20	5	(25.0%)	4	1	(25.0%)	1	0	(0.0%)	0	-	-	−25.0%	.57	-	-
Multiracial	12	5	(41.7%)	2	1	(50.0%)	4	2	(50.0%)	0	-	-	8.3%	.77	-	-
Missing Race/Ethn	96	51	(53.1%)	32	14	(43.8%)	10	4	(40.0%)	5	2	(40.0%)	−13.1%	.43	−3.8%	.87

## Glossary

Cognitive Processing Therapy: A structured, trauma-focused therapy for PTSD that helps individuals reframe and modify unhelpful thoughts related to trauma.
Prolonged Exposure: A trauma-focused therapy that reduces PTSD symptoms by gradually exposing individuals to trauma-related memories, feelings, and situations.
Sequenced Psychotherapy: A PTSD treatment approach in which patients receive non-trauma-focused psychotherapy before transitioning to trauma-focused therapy.
Suicidal Ideation: Thoughts, preoccupations, or planning related to suicide, ranging from passive considerations to active intent.
Trauma-focused Psychotherapy: A form of therapy that directly addresses the impact of trauma using evidence-based techniques like CPT, PE, or EMDR to reduce distress and improve functioning.
